# Autonomous Power Decision for the Grant Free Access MUSA Scheme in the mMTC Scenario

**DOI:** 10.3390/s21010116

**Published:** 2020-12-27

**Authors:** Wissal Ben Ameur, Philippe Mary, Jean-François Hélard, Marion Dumay, Jean Schwoerer

**Affiliations:** 1 INSA Rennes, CNRS, IETR, University Rennes, 35000 Rennes, France; Philippe.Mary@insa-rennes.fr (P.M.); Jean-Francois.Helard@insa-rennes.fr (J.-F.H.); 2 Orange Labs, 38240 Meylan, France; marion.dumay@orange.com (M.D.); jean.schwoerer@orange.com (J.S.)

**Keywords:** non-orthogonal multiple access (NOMA), multi-user shared access (MUSA), successive interference cancellation (SIC), grant free access, bit error probability (BEP), power allocation, multi-armed bandit (MAB) algorithms

## Abstract

Non-orthogonal multiple access schemes with grant free access have been recently highlighted as a prominent solution to meet the stringent requirements of massive machine-type communications (mMTCs). In particular, the multi-user shared access (MUSA) scheme has shown great potential to grant free access to the available resources. For the sake of simplicity, MUSA is generally conducted with the successive interference cancellation (SIC) receiver, which offers a low decoding complexity. However, this family of receivers requires sufficiently diversified received user powers in order to ensure the best performance and avoid the error propagation phenomenon. The power allocation has been considered as a complicated issue especially for a decentralized decision with a minimum signaling overhead. In this paper, we propose a novel algorithm for an autonomous power decision with a minimal overhead based on a tight approximation of the bit error probability (BEP) while considering the error propagation phenomenon. We investigate the efficiency of multi-armed bandit (MAB) approaches for this problem in two different reward scenarios: (i) in Scenario 1, each user reward only informs about whether its own packet was successfully transmitted or not; (ii) in Scenario 2, each user reward may carry information about the other interfering user packets. The performances of the proposed algorithm and the MAB techniques are compared in terms of the successful transmission rate. The simulation results prove that the MAB algorithms show a better performance in the second scenario compared to the first one. However, in both scenarios, the proposed algorithm outperforms the MAB techniques with a lower complexity at user equipment.

## 1. Introduction

The future radio access network of the fifth-generation is expected to support a variety of applications with different qualities of service (QoSs). These services are classified by the International Telecommunications Union and the Third-Generation Partnership Project into three main use cases with different stringent requirements, namely enhanced mobile broadband (eMBB), ultra reliable and low latency communications (uRLLC), and massive machine-type communications (mMTCs). The latter is also known as massive IoT as it is designed to mainly deal with a massive number of connected devices [1], i.e., one million connected devices per km${}^{2}$. The mMTC use case is characterized by short packet communications, i.e., on the order of a few bytes, low system complexity, and low energy consumption, which leads to a battery life on the order of ten years. The conventional orthogonal multiple access (OMA) schemes are limited by the restricted number of available orthogonal resources, and thereby, they may not be suitable to handle the huge number of devices to be connected in the mMTC scenario. However, the non-orthogonal multiple access (NOMA) schemes have been underlined as a prominent solution to address the connectivity issue [2]. In fact, they allow multiple users to simultaneously and non-orthogonally share the same resources, which increases the system overload.

In the existing technologies, users used to go through a contention-based random access protocol for data transmission. For the LTE/LTE-A network, the eNB initially broadcasts information about the available physical random access channel (PRACH) to all users. Then, each user launches a coordination process over the PRACH to ensure its alignment with the eNB. After that, for each transmission attempt, each user should send a grant acquisition request to the eNB to reserve its resource. The coordination random access channel (RACH) process is performed through four handshake steps [3]: (1) the preamble transmission; (2) the random access response; (3) the radio resource control (RRC) connection request; and (4) the RRC connection setup. However, the RACH and resource allocation processes may be very expensive in terms of signaling overhead, especially for mMTC devices.

According to [4], the transmission of 100 bytes of useful data in the uplink while going through the RACH process, security procedures, and connection release generates a signaling overhead of 59 bytes on the uplink and 136 bytes on the downlink. This induces an excessive waste of resource, a high energy consumption, and thus, a shorter battery life for the transmission of small packets. Moreover, the very high number of devices may lead to unacceptable high latency for certain mMTC applications. In fact, a large number of simultaneous connections may imply the overuse of the resources and increase the decoding error probability. For instance, under ideal system conditions, the RACH process induces a latency of 9.5 ms, which would increase significantly in the case of collision [3]. As a consequence, the random radio resource access strategy may be a performance bottleneck in some mMTC scenarios.

In this context, NOMA with the grant free access option has gained much interest, and it has been promoted by the scientific community as a promising solution to support mMTC scenarios with a minimum signaling overhead, which ensures a low energy consumption. The authors in [5] presented the evolution steps towards the uplink NOMA schemes combined with the grant free access. They suggested two possible communication scenarios for grant free access in the uplink. Users can either go with RACH-based with grant free transmission or RACH-less with grant free transmission. In the first scenario, the RACH process allows one to establish a connection with the base station and ensure user synchronizations. Then, each user transmits its data without waiting for the allocated resources from the base station. This option has never been possible for OMA schemes since granting free access may yield a severe system congestion when users transmit on the same resources. In the second scenario, users transmit their data without any beforehand communication with the base station, which significantly minimizes the signaling overhead, but at the cost of non-synchronized communications. Therefore, robust multi-user detection (MUD) receivers are required for signal detection.

Since the announcement of the advent of 5G, several NOMA schemes have emerged during the last few years, namely power domain NOMA (PD-NOMA) [6], sparse code multiple access (SCMA) [7], multi-user shared access (MUSA) [8], and pattern division multiple access (PDMA) [9], to cite a few. These schemes are different multiplexing techniques based on different keys such as the user codebook, power, or multiple domains. The authors in [10] aimed at handling the critical transmission latency issue for vehicle-to-vehicle services through a grant free access option with NOMA schemes. Two novel algorithms known as hyper-fraction and genetic algorithms were proposed to respectively reduce the system latency and improve the system throughput while guaranteeing a rate fairness between users. In [11], the authors dealt with asynchronous transmissions due to granting free access. In order to improve the decoding process, multiple copies of the same message are transmitted and then used at the receiver with the successive interference cancellation (SIC) technique as a kind of user diversity. The authors proposed closed-form expressions of the successful transmission probability, the battery lifetime, and the energy efficiency. The proposed approach may be useful for short packet communications, but at the cost of a complex decoding process. In addition, one problem of grant free access is the estimation of the number of active users. This issue was addressed in [12] by proposing a deep learning algorithm, which uses the recorded user activities at the base station to predict their future behavior. This prediction is given as an input to a modified orthogonal matching pursuit algorithm to improve the multi-user detection and reduce the error probability. In [13], a sinusoidal code was proposed for the signals’ separation in the context of the mMTC scenario with grant free access. The proposed spreading sequences permit using non-iterative algorithms for multi-user detection without prior knowledge of the channel state information and the number of active users. The authors in [14] dealt with the problem of packet collisions in a grant free access context without a re-transmission opportunity. A novel grant free access framework was proposed where the non-decoded users considered the collisions occurring as interference. Moreover, the system performance was evaluated analytically, and the authors provided simplified expressions of the outage probability and the system throughput.

SCMA has particularly been studied with grant free access protocols. For instance, in [15], the authors studied the application of SCMA with a faster than Nyquist signaling, which improved the spectral efficiency, but at the expanse of a higher inter-symbol and inter-user interference. Therefore, a novel algorithm based on the expectation propagation was proposed for the channel estimation, the detection of user activities, and the signal decoding. The work in [16] investigated an iterative message passing algorithm for grant free access SCMA, based on the belief propagation. The proposed algorithm permits jointly estimating the channel coefficients, identifying the number of active users, and detecting the transmitted data while improving the bit error rate compared to the other techniques.

Regarding the system design, MUSA has the potential to enable grant free access with minimum signaling overhead in the context of mMTC applications. Unlike the SCMA scheme, which requires the assignment of the codebook beforehand, in MUSA, each user randomly and autonomously selects a spreading sequence within a predefined constellation. In other words, users can transmit their data at any moment without going through a resource allocation process with the base station, which minimizes the amount of signaling overhead. The MUSA scheme is typically used with a SIC receiver for multi-user detection, which provides a low decoding complexity. However, the SIC technique may suffer from the error propagation phenomenon when the received powers are similar [17]. The power allocation process is usually performed in a centralized manner [18,19] where the base station knows the channel state information of all users. For grant free access, each user performs a blind transmission with no information about its propagation environment and interfering users, which makes the power determination more complex.

The autonomous power decision for NOMA schemes with the grant free access strategy has recently been investigated in several works. An interesting solution is to use multi-armed bandit (MAB) algorithms, which belong to the global reinforcement learning paradigm [20,21]. MAB techniques can be applied to the problem of dynamic resource allocation by balancing between the exploration and exploitation phases. At each time, each agent selects an arm, i.e., representing the physical resource to be shared, among a set according to a predefined policy in order to maximize its cumulative reward and hence minimize its regret. The MAB algorithms have been used in several applications such as marketing, advertising, and cellular communications. For instance, the authors in [22] applied the MAB algorithms to the autonomous power decision problem in order to maximize the user rates for the PD-NOMA scheme. The user rewards are their rates. However, these may be carried on many bits, which increases the signaling overhead; hence, it may not really be adapted for mMTC scenarios. MAB was also merged with NOMA schemes in [23] where the authors proposed a distributed NOMA-based MAB approach to handle the channel access problem in cognitive radio networks. Moreover, the authors in [24] performed the MAB algorithms in the LTE cellular network for an autonomous subcarrier allocation in a dense network while taking into consideration the dynamic resource occupation in each surrounding cell.

To the best of our knowledge, no work has investigated the problem of autonomous power decision for grant free access with MUSA scheme. The characteristics of spreading sequences and the principle of the SIC receiver make the power decision more complex. Therefore, in this paper, we deal with this issue with minimum signaling overhead to address the mMTC requirements. The goal is to improve the system performance measured with the successful transmission rate in order to achieve the performance of an optimal centralized power allocation. The latter is quite difficult to obtain, especially for SIC receivers with the error propagation problem. To do so, we start by proposing an approximated expression for the bit error probability (BEP) while considering the inter-user interference and the effect of error propagation. The optimal power value of users is obtained as the solution of the minimization of the global average BEP. Based on the derived BEP expression, we propose a novel algorithm for power selection for the MUSA scheme with a reduced signaling overhead. The proposed algorithm is compared with known index-based MAB algorithms adapted to the power selection by each user. In this part, we propose to investigate two scenarios for selecting the best arm by each MAB algorithm: a scenario where the arm index computation by a user is only based on the decoding status of its own packet, i.e., success or failure, and another scenario where it depends on the decoding status of the other users’ packets in addition to its own packet decoding status.

This paper is organized as follows. The system model and the fundamentals of MUSA are introduced in Section 2. The SIC receiver is revisited in Section 3, while a closed-form expression for users’ bit error probability is derived in Section 4. Then, the proposed algorithm for autonomous power decision is described in Section 5. The multi-armed bandit algorithms and the studied scenarios are introduced in Section 6. A comparison of all power decision approaches is provided in Section 7. Numerical results and performance analysis are conducted in Section 8, and conclusions are drawn in Section 9.

Notations: Vectors and matrices are denoted in lowercase and uppercase, respectively, and in bold font, while scalars use a normal font weight. The complex and real number sets are denoted by C and R, respectively. Moreover, ${\left(.\right)}^{T}$ and ${\left(.\right)}^{H}$ stand for the transpose and Hermitian operations. diag(a) represents the diagonal matrix created with the elements of vector a in the main diagonal.

## 2. System Model

**Figure 1:**
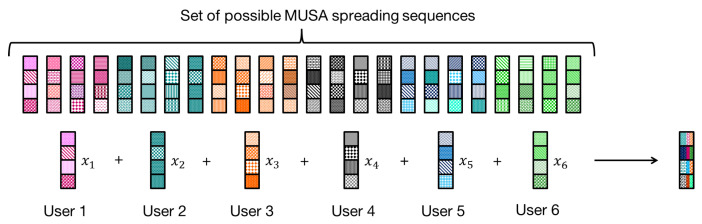
MUSA scheme system for J=6 and K=4.

An uplink communication system of *J* users transmitting over *K* orthogonal subcarriers is considered. The active users share the available resources using the MUSA scheme with grant free access. Each user’s bits are mapped to a series of symbols through an M-ary modulation block. Then, the modulated symbols are multiplied by the users spreading sequences and spread over the available subcarriers, as illustrated in Figure 1. User sequences $s_{j}$, $\forall j\in \left\{1,\cdots ,J\right\}$ are such that $s_{j}\in {\left\{a+jb\right\}}^{K}$, where $\left(a,b\right)\in {\left\{-1,0,1\right\}}^{2}$. The received signal on subcarrier *k* of each OFDM symbol is:

(1) $$y_{k}=\sum_{j=1}^{J}\sqrt{p_{j}}h_{kj}s_{kj}x_{j}+n_{k}$$

where $h_{kj}$ and $s_{kj}$ are the *k*-th component of the *j*-th user channel vector and spreading sequence, i.e., $h_{j}$ and $s_{j}$, respectively. Moreover $x_{j}$, $p_{j}$ are the transmitted symbol and the transmission power of the *j*-th user, respectively, and $n_{k}$ is the additive white Gaussian noise component on the *k*-th subcarrier with $n∼\mathrm{CN}(0,\sigma^{2}I_{K})$, where $I_{K}$ is the *K*-by-*K* identity matrix. The multiplexed received signals on all subcarriers can be written as:

(2) $$y=GP^{\frac{1}{2}}x+n$$

where $P=\mathrm{diag}\left(p_{1},p_{2},\ldots ,p_{J}\right)\in R_{+}^{J\times J}$ is the transmission power matrix, $x={\left[x_{1},x_{2},\ldots ,x_{J}\right]}^{T}$ is the transmitted users’ symbols with $E\left[xx^{H}\right]=I_{J}$, and G is the equivalent channel matrix including the spreading sequences such that:

(3) G=H⊙S

where $H=\left[h_{1},\cdots ,h_{J}\right]$, $S=\left[s_{1},\cdots ,s_{J}\right]$, and ⊙ is the Hadamard product, i.e., $g_{kj}=h_{kj}s_{kj}$.

## 3. Multi-User Detection

The SIC receiver offers a low decoding complexity compared to other MUD algorithms, namely the message passing algorithm or maximum a posteriori algorithm [25]. However, SIC’s performance depends on the user received powers, and the receiver performs better when the received powers are sufficiently different. MUSA is typically used with ordered-SIC jointly with a linear detection receiver such as the minimum mean squared error (MMSE). The MMSE matrix is calculated as in [26]:

(4) $$W^{H}={\left(P^{\frac{1}{2}}G^{H}GP^{\frac{1}{2}}+\sigma^{2}I\right)}^{-1}P^{\frac{1}{2}}G^{H}.$$

The main principle of the ordered-SIC technique is to successively estimate the user symbol, reconstruct the generated interference, and then, subtract it from the received signal. User symbols are decoded in a descending order of their SINRs. Assume that the received signal at the *j*-th iteration is:

(5) $$y^{j}=\sqrt{p_{j}}g_{j}x_{j}+\sum_{i=j+1}^{J}\sqrt{p_{i}}g_{i}x_{i}+n^{j},$$

where $g_{j}$ is the *j*-th column of the matrix G. Then, the SINR of the user *j* picked to be decoded is:

(6) $$\beta_{j}\left(p\right)=\frac{p_{j}{\left|w_{j}^{H}g_{j}\right|}^{2}}{\sum_{i=j+1}^{J}p_{i}|w_{j}^{H}g_{i}{|}^{2}+\sigma^{2}{∥w_{j}^{H}∥}^{2}},$$

where $w_{j}$ is the *j*-th column of the MMSE matrix W. After that, the user symbol is estimated by multiplying the row vector $w_{j}^{H}$ by the received column signal as follows:

(7) $${\hat{x}}_{j}=w_{j}^{H}y.$$

The interference generated by the *j*-th user is reconstructed and then subtracted from the received signal, which is updated as follows:

(8) $$y=y-g_{j}{\hat{x}}_{j}.$$

After each iteration, the *j*-th column of the matrix G, corresponding to the decoded user *j*, is removed, and the MMSE matrix is recalculated as in (4). This process is repeated until all users are decoded.

## 4. BEP Analysis

The error propagation is one of the critical issue of SIC receivers, which significantly deteriorates the system performance and makes the derivation of the BEP expression more complicated. For a Gray mapping, two adjacent symbols are different in only one single bit. Hence, assuming the inter-user interference as noise, the erroneous detection often leads to the detection of an adjacent symbol with only one wrong bit compared to the correct symbol [27]. Therefore, the average system BEP is well approximated as:

(9) $$P_{b,\mathrm{MMSE}-\mathrm{SIC}}\approx \frac{1}{J\log_{2}\left(M\right)}\sum_{j=1}^{J}P_{ej}$$

where $P_{ej}$ is the symbol error probability (SEP) of the *j*-th user. In the following, we investigate the BEP of the MMSE-SIC receiver with two different hypotheses: (i) perfect SIC with no error propagation (NEP); (ii) imperfect SIC with error propagation (EP).

### 4.1. Perfect SIC without Error Propagation

In this case, since there is no error propagation in the receiver, the BEP is calculated similarly as for the MMSE receiver while updating the MMSE matrix at each iteration, and the SINRs are calculated as in (6). For a QPSK modulation and assuming the inter-user interference as noise [28], the *j*-th user SEP is approximated as [27]:

(10) $$\begin{array}{cc} \; & P_{ej}\approx 2Q\left(\sqrt{\beta_{j}^{\mathrm{NEP}}\left(p\right)}\right)\left(1-0.5Q\left(\sqrt{\beta_{j}^{\mathrm{NEP}}\left(p\right)}\right)\right). \end{array}$$

### 4.2. Imperfect SIC with Error Propagation

In that case, the BEP of each user depends on the previously decoded users. In this paper, we were inspired by the proposed approach in [28], and thereby, the SEP of the *j*-th user is calculated as:

(11) $$P_{\varepsilon_{j}}=\sum_{i=0}^{N_{j}-1}P\left\{\varepsilon_{j}|b_{i}^{j}\right\}P\left\{b_{i}^{j}\right\},$$

where $N_{j}=2^{j-1}$ is the number of possible (j−1)-dimensional binary sequences and $b_{i}^{j}=\left(b_{i,1}^{j},b_{i,2}^{j},\cdots ,b_{i,j-1}^{j}\right)$$\forall i\in \left\{0,\cdots ,N_{j}-1\right\}$ and $j\in \left\{1,\cdots ,J\right\}$, with $b_{i,k}^{j}=0$ if the symbol of the *k*-th decoded user is correctly detected and one otherwise. Each sequence refers to the state, correctly decoded or not, of all the previously (j−1) decoded users. The event $\varepsilon_{j}$ indicates an erroneous detection of the *j*-th user symbol. Hence, $P\left\{\varepsilon_{j}|b_{i}^{j}\right\}$ is the error probability of the *j*-th user symbol conditioned on the sequence $b_{i}^{j}$. Considering an eventual error propagation occurrence, the received signal at the *j*-th SIC iteration is represented as:

(12) $$y^{j}=\sqrt{p_{j}}g_{j}x_{j}+\sum_{i=j+1}^{J}\sqrt{p_{i}}g_{i}x_{i}+\sum_{k=1}^{j-1}\sqrt{p_{k}}g_{k}\left(x_{k}-{\hat{x}}_{k}\right)+n^{j},$$

where ${\hat{x}}_{k}$ is the faulty estimation of $x_{k}$. The additional term compared to (5) is generated by the erroneous detection of the previous users. This may significantly affect the system performance. Therefore, the experienced noise and the new interference term can be combined in $n_{eq}=\sum_{k=1}^{j-1}\sqrt{p_{k}}g_{k}\left(x_{k}-{\hat{x}}_{k}\right)+n^{j}$. The resulting term is approximated as a centered Gaussian random variable, where $E\left\{n_{eq}\right\}=0$ and $E\left\{n_{eq}n_{eq}^{H}\right\}=\left(\sum_{k=1}^{j-1}p_{k}{∥g_{k}∥}^{2}E\left\{∥x_{k}-{\hat{x}}_{k}{∥}^{2}\right\}+\sigma^{2}\right)I=\left(\sum_{k=1}^{j-1}p_{k}{∥g_{k}∥}^{2}\delta_{k}d+\sigma^{2}\right)I$. We define *d* as the square of the euclidean distance between the neighboring symbols and $\delta_{k}=1$ if $x_{k}\neq {\hat{x}}_{k}$ and zero otherwise. As a consequence, the SINR of the *j*-th user, corresponding to the detection combination $b_{i}^{j}$, is calculated as follows:

(13) $$\beta_{j,i}^{\mathrm{EP}}\left(p\right)=\frac{p_{j}{\left|w_{j}^{H}g_{j}\right|}^{2}}{\sum_{i=j+1}^{J}p_{i}|w_{j}^{H}g_{i}{|}^{2}+\left(\sum_{k=1}^{j-1}p_{k}{∥g_{k}∥}^{2}\delta_{k}d+\sigma^{2}\right){∥w_{j}^{H}∥}^{2}}$$

Two main terms should be calculated to obtain the user SEP. Starting by the conditional probability, which is calculated according to (10) and (13), we have:

(14) $$\begin{array}{cc} \; & P\left\{\varepsilon_{j}|b_{i}^{j}\right\}=2Q\left(\sqrt{\beta_{j,i}^{\mathrm{EP}}\left(p\right)}\right)\left(1-0.5Q\left(\sqrt{\beta_{j,i}^{\mathrm{EP}}\left(p\right)}\right)\right). \end{array}$$

However, the probability of the combination $b_{i}^{j}$ is readily calculated as:

(15) $$\begin{array}{cc} P\left\{b_{i}^{j}\right\}=P\left\{\cap_{n=1}^{j-1}b_{i,n}^{j}\right\} & =\prod_{n=1}^{j-1}P\left\{b_{i,n}^{j}\cap_{m=1}^{n-1}b_{i,m}^{j}\right\}, \end{array}$$

where $P\left\{b_{i,n}^{j}|\cap_{m=1}^{n-1}b_{i,m}^{j}\right\}$ is the probability that the *n*-th symbol is correctly decoded or not, i.e., $b_{i,n}^{j}=0$ or $b_{i,n}^{j}=1$, conditioned on the estimation of the previously decoded (n−1) symbols. It is calculated as:

(16)Pbi,nj|∩m=1n−1bi,mj=(17)1−2Qβn,iEP(p)1−0.5Qβn,iEP(p)ifbi,nj=02Qβn,iEP(p)1−0.5Qβn,iEP(p)otherwise.

For an uplink transmission, devices are restricted by a maximum transmission power, $p^{U}$, imposed by the regulation authorities and the equipment design restrictions. Therefore, an optimal centralized power allocation $p_{\mathrm{opt}}$ that minimizes the global average error probability can be obtained by solving the following problem:

OP1{(18a)minp1Jlog2(M)∑j=1J∑i=0Nj−1P{εj|bij}P{bij}(18b)pj≤pU    ∀j∈J

where J={1,2,⋯,J} is the set of active users. The derived expression of the user SEP is quite complicated to analyze theoretically with the Karush–Kuhn–Tucker (KKT) conditions. Therefore, we use an advanced optimization algorithm, i.e., particle swarm optimization [29], to solve the power allocation problem above. This algorithm is known to be efficient for complex problems [30].

## 5. Proposed Autonomous Power Decision Algorithm

Each user has to decide its transmission power autonomously with no information about the propagation environment and the interference. In this section, we aim at proposing an autonomous power decision algorithm for uplink communication. This allows each user to select an adequate power value close to the optimal one, $p_{\mathrm{opt}}$, obtained by solving $OP_{1}$.

The key idea is to perform an iterative algorithm that takes advantage of the natural base station acknowledgment (ACK). Each user gradually updates its transmitted power from the received ACK in order to converge toward the nearest power level from $p^{\mathrm{opt}}$. For example, the *j*-th user initially transmits its data with a randomly selected power $p^{j}$ within the interval $\left[p_{\min}^{j},p_{\max}^{j}\right]$, where $p_{\min}^{j}$ and $p_{\max}^{j}$ are respectively the initial minimum and maximum power values memorized in the *j*-th user equipment (UE). Then, the base station detects the user signal and compares its transmission power with $p^{j,\mathrm{opt}}$, which the base station has computed on its own. An acknowledgment will be sent back to each user to adjust its power. In order to minimize the signaling overhead, the acknowledgment is carried on two bits and can hence encode four possible states: (1) ACK=3 if the user should simply transmit with its maximum authorized power $p^{U}$; this case may be gainful for the cell edge users that experience bad propagation conditions; (2) ACK=2 if $p^{j}>p^{j,\mathrm{opt}}$; (3) ACK=1 if $p^{j}<p^{j,\mathrm{opt}}$; and (4) ACK=0 if $p^{j}=p^{j,\mathrm{opt}}$. Each user updates its interval by shifting the $p_{\min}^{j}$ and $p_{\max}^{j}$ values. After that, it picks up another random value in the new power interval for the next packet transmission until it arrives at the appropriate power value. However, the channel conditions may change along the way. Hence, the algorithm must take this into consideration in order to ensure its convergence and assure the best performance. For that reason, the base station may, sometimes, send another extra bit “Stat” to notify the user of this occurrence. In this case, the UE will try to initialize its power interval while taking advantage of the previously sent packets. This process is described in detail in Algorithm 1.
**Algorithm 1:** Autonomous power decision.**Require:**$p_{\max}^{j}=p^{U}$, $p_{\min}^{j}>0$∀j=1,2,⋯,J;**Ensure:**p 1:Each user picks up its spreading sequences. 2:Each user selects a random power level $p^{j}\in \left[p_{\min}^{j},p_{\max}^{j}\right]$. 3:The BS detects user signals. 4:The BS calculates the optimal power $p^{j,\mathrm{opt}}$. 5:The BS compares each user power $p^{j}$ with the nearest power level from $p^{j,\mathrm{opt}}$. 6:The BS sends an acknowledgment to each user: (a)If $p^{j,\mathrm{opt}}=p^{u}$⇒ACK=3 (b)If $p^{j}>p^{j,\mathrm{opt}}$⇒ACK=2 (c)If $p^{j}<p^{j,\mathrm{opt}}$⇒ACK=1 (d)If $p^{j}=p^{j,\mathrm{opt}}$⇒ACK=0 7:If the propagation environment is changed, the BS sends a one bit ACK: Stat=1. 8:Each user updates its $p_{\min}^{j}$ or $p_{\max}^{j}$:
 (a)If ACK=3⇒$p^{j}=p^{u}>0$ (b)If ACK=2⇒$p_{\max}^{j}=p^{j}\leq p^{U}$
If Stat=1⇒$p_{\min}^{j}=0$ (c)If ACK=1⇒$p_{\min}^{j}=p^{j}>0$
If Stat=1⇒$p_{\max}^{j}=p^{U}$ (d)If ACK=0⇒ no update 9:Return to Step 2

The channel should not change too fast in order to allow the convergence of the algorithm. However, as will be seen in the simulation results, the proposed algorithm converges to the near-optimal power value quite quickly. In addition, users’ transmission powers must be known at the BS to perform the proposed algorithm. However, these power values are obviously needed in order to apply the SIC receiver properly. Therefore, a calibration phase between the BS and the UE should always be established.

## 6. Power Allocation with Multi-Armed Bandits

In this section, we revisit three known MAB algorithms, i.e., ϵ-greedy, upper confidence bound (UCB1), and Thompson sampling (THS), that we apply to our autonomous power selection problem. An MAB is a model with *N* resources, called arms, each of them being associated with a reward following a specific probability distribution. At each time slot *t*, each agent *j* plays an arm $a_{j}$ according to its policy. Then, it receives the corresponding reward $r_{j}^{t}\left(a_{j}\right)$. Based on this and the number of times each arm has been played so far, $n_{j}^{t}\left(a_{j}\right)$, each agent chooses the appropriate arm for the next time slot t+1, according to the calculated index that depends on each algorithm policy. Over time, these techniques will prioritize the arms showing the best performance and exclude the worst ones.

All MAB algorithms search for the maximization of the cumulative rewards of each agent over the time horizon *T*, i.e., $\sum_{t=1}^{T}r_{j}^{t}\left(a_{j}\right)$ and thereby the minimization of its regret $R_{j}$ defined as the difference between the rewards obtained using the chosen policy and the expected reward we would obtain if the best arm were always played, i.e., $r_{j}^{*}$. The *j*-th user regret during a maximum period of *T* slots is calculated as follows:

(19) $$R_{j}=Tr_{j}^{*}-\sum_{t=1}^{T}E\{r_{j}^{t}\left(a_{j}\right)\}$$

In our case, we consider a multi-agent system where the agent refers to the UE and the arms represent the power levels. At the *t*-th iteration, the successful transmission rate of the *j*-th user is defined as the ratio between the cumulative number of its correctly received packets during *t* time slots and the total number of plays so far. The MAB algorithms are investigated in two different scenarios detailed hereafter.

(a) Scenario 1:

The base station acknowledgment at the *t*-th iteration is carried on 1 bit representing the corresponding user reward, i.e., $r_{j}^{t}\in \left\{0,1\right\}$. At each time slot *t*, $r_{j}^{t}\left(a_{j}\right)=1$ if the packet of the *j*-th user is successfully decoded and $r_{j}^{t}\left(a_{j}\right)=0$ otherwise. Therefore, the successful transmission rate of the *j*-th user at the *t*-th iteration is calculated as $Q_{j}^{t}=\frac{\sum_{i=1}^{t}r_{j}^{i}\left(a_{j}\right)}{t}$. In this scenario, the reward of each user only depends on the decoding status of its own packet without any consideration of the other users. However, the successful decoding event of one packet depends on the successful decoding of the others, because of the SIC receiver. Hence, every user has interest in good power selection for the other users and not only for itself. Scenario 2, we propose hereafter, takes into account this fact.

(b) Scenario 2:

The base station acknowledgment at the *t*-th iteration is now carried on two bits $\left\{b_{2,j}^{t}b_{1,j}^{t}\right\}$. The first bit informs whether all users are correctly decoded, $b_{1,j}^{t}=1$, or, at least, one packet is erroneously detected, $b_{1,j}^{t}=0$. The second bit notifies each user whether its own packet is correctly received, $b_{2,j}^{t}=1$, or not, $b_{2,j}^{t}=0$. For a picked power $p_{j}$ by user *j*, there are three possible states for the j-th user acknowledgment $\left\{b_{2,j}^{t}b_{1,j}^{t}\right\}\in \left\{11,10,00\right\}=\left\{3,2,0\right\}$. The case where $\left\{b_{2,j}^{t}b_{1,j}^{t}\right\}=01$ is not possible because $b_{1,j}=1$ means that all packets have been correctly decoded, including the *j*-th user packet, and hence, $b_{2,j}$ is automatically equal to one. In order to meet the conditions of the convergence theorems derived in [31], the rewards should be supported in [0,1]. Therefore, user rewards are defined as a normalization of the associated acknowledgments, i.e., $r_{j}^{t}\in \left\{1,\frac{2}{3},0\right\}$. The successful transmission rate, at the *t*-th iteration, of the *j*-th user is then calculated based only on the second bit $b_{2,j}^{t}$, i.e., $Q_{j}^{t}=\frac{\sum_{i=1}^{t}b_{2,j}^{i}}{t}$. In this scenario, the inter-user dependence is involved in the associated rewards.

### 6.1. UCB1

UCB1 was inspired by the Agrawal’s index-based policy [31]. This algorithm has a uniformly logarithmic regret over time. Generally, the UCB family of algorithms relies to a confidence interval on the average reward of each arm [32]. the UCB1 index gathers two functions; the average reward and the exploration term. This index refers to an estimation of the upper bound of the true expectation of the arm reward. It is an upper bound because the square root term is an estimation of the variance of the expected return when playing the arm $a_{j}$ and is defined as follows, at time slot *t*:

(20) $$\frac{1}{n_{j}^{t}\left(a_{j}\right)}\sum_{i=1}^{t}r_{j}^{i}\left(a_{j}\right)+\sqrt{\frac{\theta \log(t)}{n_{j}^{t}\left(a_{j}\right)}}$$

where θ>0 is the exploration parameter. Originally, UCB1 was proposed with θ=2; however, the authors in [32] mentioned that θ=0.5 performs better empirically although θ>0.5 is strongly recommended for the theoretical analysis.

At the initialization phase, UCB1 explores each arm once in order to have an estimation of the reward of each arm. Then, at each iteration, each user selects the arm with the highest index, as illustrated in Algorithm 2. The calculated index (20) ensures the balance between the exploration of the most uncertain arms and the exploitation of the best arm so far. UCB1 prescribes the principle of “optimism in the face of uncertainty”, which means that the less visited arm seems more uncertain, and thereby, it may optimistically be the best arm to play.
**Algorithm 2:** UCB1 algorithm.
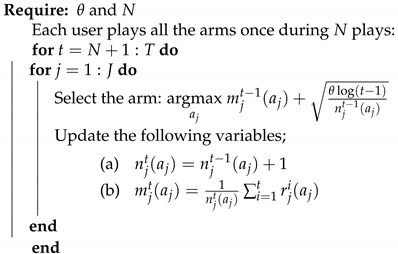


### 6.2. ϵ-Greedy

This algorithm deals with the exploration and the exploitation dilemma randomly. At each iteration, each user either explores arbitrarily a new arm with probability ϵ or it plays the best arm corresponding to the highest average reward so far with a probability of 1−ϵ. However, for a constant exploration parameter ϵ, the system regret evolves linearly overtime instead of being logarithmic. On the one hand, for a high ϵ value, i.e., ϵ≈1, the user will continue to only explore random arms even if it came out with the best arm, and on the other hand, for a low ϵ value, i.e., ϵ<<1, the algorithm will tend to exploit all the time even if it has not sufficiently explored the other arms. In both cases, an important performance loss will be experienced. Therefore, the ϵ value is a critical parameter. A revised version called ϵ-decreasing greedy has been proposed, where the exploration probability is decreasing toward zero over time with a rate of $\frac{1}{t}$. This allows one to essentially explore at the beginning of the learning and mostly to exploit the best arm found so far after a certain amount of time. The new exploration probability is defined as [22,31]:

(21) $$\epsilon \left(t\right)=\min\left\{1,\frac{CN}{d^{2}t}\right\}\overset{△}{=}\min\left\{1,\frac{LN}{t}\right\}.$$

where L>0 is the exploration parameter. However, the main challenge of this policy is how to properly set the value of *L*. The ϵ-decreasing greedy algorithm is described in detail in Algorithm 3.
**Algorithm 3:**ϵ-decreasing greedy algorithm.
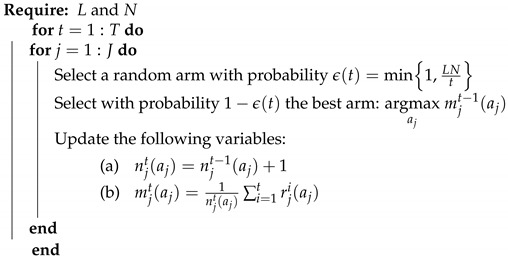


### 6.3. Thompson Sampling Algorithm

This approach shows a robust performance for stochastic problems and sometimes outperforms other MAB algorithms. The THS algorithm belongs to the Bayesian MAB family. The *j*-th user starts by a uniform prior beta distribution $\beta (\alpha_{j,k},\gamma_{j,k})$ for all arms with initial values $\alpha_{j,k}=\gamma_{j,k}=2$∀j∈{1,⋯,J} and ∀k∈{1,⋯,N}, where *k* refers to the arm index among *N* power levels. Then, inspired by the case where rewards follow a binomial distribution [33] and based on the observed reward, the parameters of the posterior beta distribution are updated such that $\alpha_{j,k}=\alpha_{j,k}+3r_{j}^{t}$ and $\gamma_{j,k}=\gamma_{j,k}+3\left(1-r_{j}^{t}\right)$.

At the next time slot, each user draws a sampled index from the updated beta distribution for each arm, i.e., $i_{j,k}∼\beta \left(\alpha_{j,k},\gamma_{j,k}\right)$∀k∈1,⋯,N and ∀j∈1,⋯,J. The arm with the highest index, i.e., ${\hat{i}}_{j,k}=\max_{k\in N}\left(i_{j,k}\right)$∀j∈1,⋯,J, is hence elected for this transmission attempt. Through time, Thompson sampling prioritizes the arm with the highest probability of being the optimal one and avoids other arms that have demonstrated poor performance so far. This algorithm is described in detail in Algorithm 4.
**Algorithm 4:** Thompson sampling algorithm.
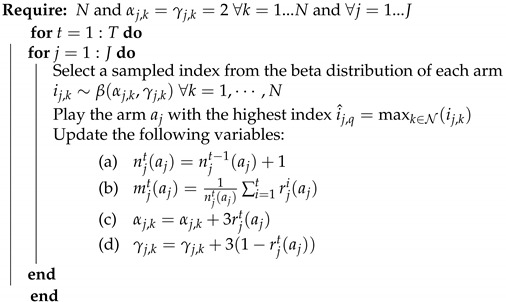


## 7. Complexity and Overhead Analysis

A quantitative comparison of all the examined techniques in the context of the mMTC scenario is summarized in Table 1. The random power selection and the centralized allocation are taken as reference scenarios. The centralized allocation is the reference in terms of performance, and the random selection is the simplest one.

The centralized power allocation algorithm computes, at the base station, the power to allocate to the users at each transmission attempt, based on the users’ received SINRs. All the complexity is located at the base station, and users have to set their transmitting power at the values sent back from the BS; hence, the algorithm complexity at the user side is O(1). The signaling overhead of this scheme cannot be assessed precisely since it strongly depends on the downlink control information (DCI) format. However, the power computed is quantized over *k* bits, which would likely be much larger than one or two bits, for each user. Hence, for a large number of users, the signaling would be at least in O(J·k). Thus, it may be very expensive in terms of energy consumption, leading to a significant reduction of the battery lifetime.

The random power selection does not manifest any algorithmic complexity since the power selection is realized randomly. Therefore, the generated signaling overhead is minimal, i.e., 1 bit, as it only relies on the acknowledgment sent by the BS for each user’s packet, whether it is successfully received or not.

The proposed autonomous power decision algorithm is based on four acknowledgment levels, used to update the power at the user side, which can be carried with two bits. Moreover, one may add one additional bit if the BS detects a channel variation in order to notify the corresponding user of this event. The generated complexity is on the order of O(1) as no computation is required at the UE during this process.

All the MAB techniques have the same signaling overhead and algorithmic complexity for each transmission attempt. UCB1, ϵ-decreasing greedy, and Thompson sampling can be seen as index-based policies. Hence, the algorithmic complexity consists of sorting *N* indexes, representing the rating of the arms w.r.t. the objective of the agent, and taking the arm that corresponds to the highest index. Therefore, their complexity is on the order of O(N). Furthermore, the generated signaling overhead depends particularly on the applied learning scenario. In Scenario 1, the index update by an agent is only based on the processing output of its own packet using a given power, i.e., either the packet is successfully received or not, and hence, it takes 1 bit. In Scenario 2, the update of an agent index is made by taking into account the decoding status of the other users’ transmissions, in addition to that of its own packet, which is carried out with two bits. It is worth noting that the computational complexity is not considered here. Moreover, the complexity of calculating a sampled index from the beta function for each arm with the Thompson sampling algorithm is higher than that of the UCB1 and ϵ-decreasing greedy indexes.

## 8. Numerical Results and Analysis

We consider an uplink system with 150% of overload, where J=12 and K=8. Users are uniformly scattered in the cell while experiencing an AWGN channel with different path losses. Each user can pick its transmission power over a set of N=10 possible power levels in the interest of selecting the appropriate value ensuring the best performance in both Scenarios 1 and 2. The user spreading sequences are normalized to unitary energy. The algorithms are investigated in term of the successful transmission rate, i.e., the total number of correctly decoded packets over the total number of sent packets. Simulations are averaged over 150 network realizations, i.e., the successful transmission rate is averaged over the path losses and the spreading sequences. Regarding the UCB1 algorithm, the exploration of new power values is conducted by the parameter θ. As mentioned above, this parameter was originally set to two, but in the literature, θ=0.5 is admitted empirically as it provides better performance. In order to choose the optimal value of θ, the average transmission rate achieved by UCB1 was investigated w.r.t. θ, and the value θ=0.5 is the one that allows achieving the best transmission rate. The figure is not reported here so as not to clutter the exposure. The other simulation parameters are given in Table 2.

**Figure 2:**
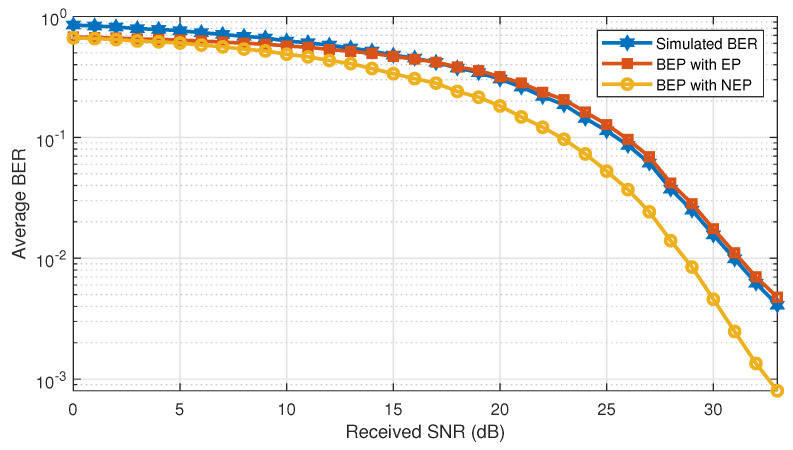
Performance comparison of the simulated BER and the analytical BEP for an AWGN channel with different users’ path loss and equal transmission powers.

Figure 2 compares the simulated average BER, i.e., averaged over the spreading sequences and positions, and the analytical average BEP obtained by the proposed expression in (9) for an AWGN channel and uniformly distributed users over the cell w.r.t. the global received SNR. We remark that the expression that takes into account the error propagation phenomenon almost matches the simulated BER. However, removing the error propagation effect induces a wide gap in the performance because it is too optimistic. In addition, we notice that, for high SNR values, the BEP with EPgets closer to the simulated BER. This can be explained by the fact that the QPSK approximation in (10) is more robust for high SNR.

**Figure 3:**
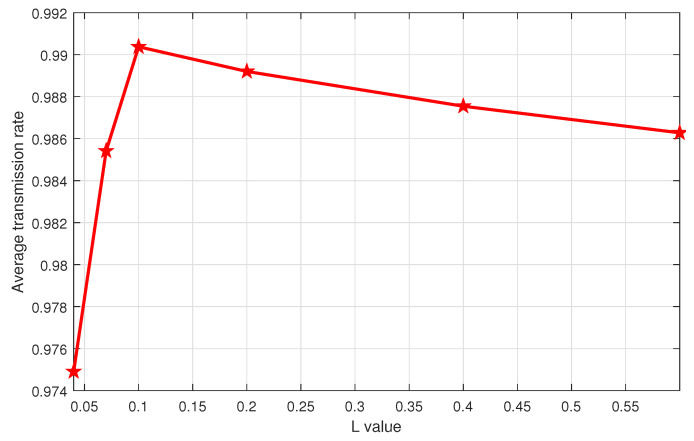
Performance comparison of ϵ-decreasing greedy for different *L* values after T=1000 iterations in Scenario 1.

The performance of the ϵ-decreasing greedy algorithm depends on the ϵ value, which in turn depends on the coefficient *L*. It is important to choose the coefficient that allows the algorithm to achieve its best performance. Therefore, the main challenge of the ϵ-decreasing greedy approach is to handle the exploration and the exploitation dilemma by properly setting the value of *L* in (21). Figure 3 investigates the performance of this algorithm for different *L* in Scenario 1 after T=1000 iterations. We note that L=0.1 gives the best performance in terms of the average transmission rate and hence it is kept for the rest of the simulations. The same behavior is observed in Scenario 2, but not reported here to limit the redundancy.

**Figure 4:**
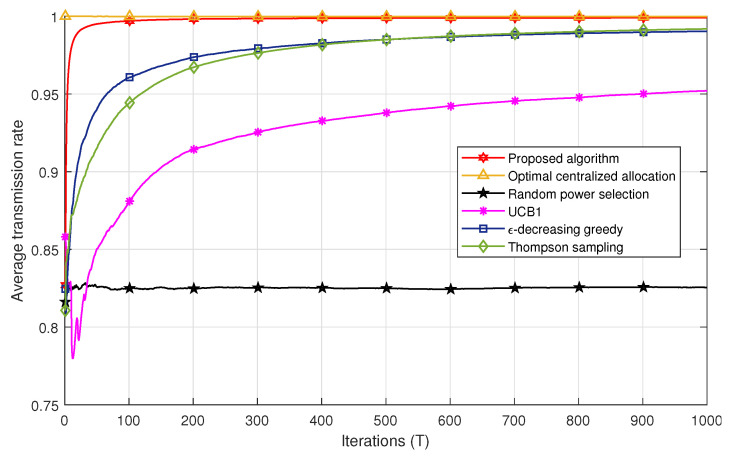
Successful transmission rate comparison for all algorithms in Scenario 1.

**Figure 5:**
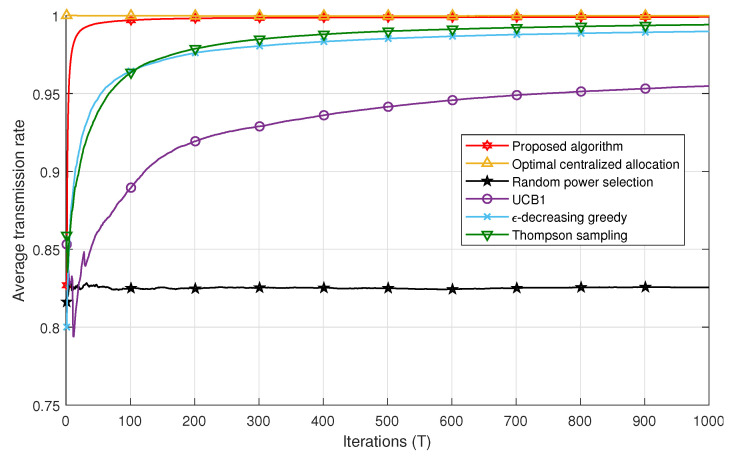
Successful transmission rate comparison for all algorithms in Scenario 2.

Figure 4 and Figure 5 compare the successful transmission rate of the algorithms under study, i.e., the centralized power allocation, the proposed algorithm, the MAB algorithms (ϵ-decreasing greedy, UCB1, and THS), and the random power selection in Scenarios 1 and 2, respectively. The proposed algorithm outperforms all the MAB techniques with a faster convergence to the optimal power in both scenarios. We also remark in Figure 4 that the ϵ-decreasing greedy algorithm converges faster than the THS and UCB1 algorithms. This can be explained by the optimal selection of the *L* value, which ensures a trade-off between the exploration and the exploitation phases in order to achieve the best performance. The ϵ-decreasing greedy and THS algorithms converge to the same successful transmission rate after 400 iterations. However, the gap between ϵ-decreasing greedy and THS is less important in Scenario 2 in Figure 5. In fact, after T=100 iterations, THS is slightly better than ϵ-decreasing greedy. THS seems to take advantage of the additional information carried by the feedback whether there is a decoding error among the users or not. However, both algorithms, i.e., ϵ-decreasing greedy and THS, are far better than UCB1 in both scenarios. UCB1 takes more time to explore suboptimal powers, which slows down its convergence to the optimal power values and thereby induces more packet losses. The random power allocation presents the lowest performance bound in both scenarios since no strategy is applied for an adequate power selection, which induces error propagation and hence packet losses.

For a given number of iterations *T*, the figures represent the average successful transmission rate achieved after averaging over the network realizations and the spreading sequences, i.e., 150 realizations, *T* being the number of packets sent, also known as the number of iterations in each algorithm. The performance achieved by the algorithms under fast variations of the propagation environment is directly obtained from Figure 4 and Figure 5 by shortening them to the desired value of *T*. In other words, if one would want to obtain the achievable successful rate of the different algorithms when the environment changes every 100 packets, then one should collect the points at T=100 in each figure above. Moreover, a fading channel could have been considered also; however, this would only affect the absolute performance, as the statistic of the rewards would have been changed, but not the relative behaviors of the algorithms. Therefore, in this paper and for the sake of simplicity, we consider only an AWGN channel with different path losses among users, and we show the behavior of the investigated techniques as the number of iterations increases averaged over several network realizations.

**Figure 6:**
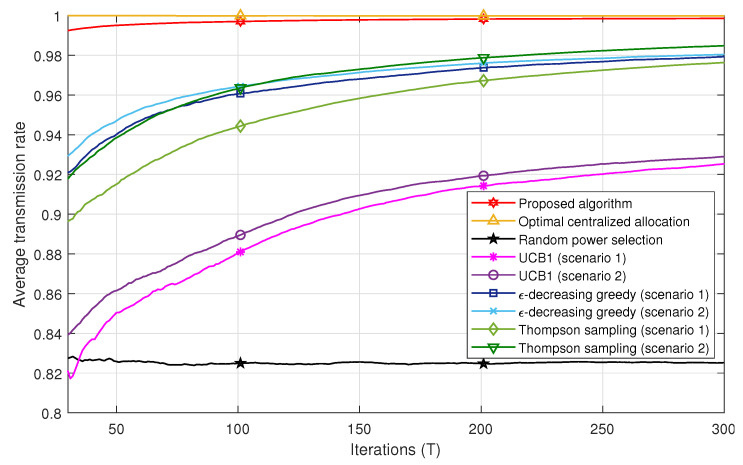
Successful transmission rate comparison for all algorithms in Scenarios 1 and 2.

Figure 6 shows the performance comparison of all algorithms in Scenarios 1 and 2 for 30≤T≤300. One can remark that all MAB techniques achieve better performances in Scenario 2 compared to Scenario 1. For instance, after T=50 iterations, the Thompson sampling algorithm achieves a successful transmission rate of ≈0.94 in Scenario 2, whereas, in Scenario 1, it attains the value of 0.91. This may be explained by the fact that Scenario 2 conveys more information compared to Scenario 1 to select the best set of powers. In other words, the reward a user gets in Scenario 2 is not only a function of the successful decoding of its own packet, but also whether all other users succeeded in their transmissions or not. This strategy allows each user to take into account a kind of global interest in the selection of its power. In addition, the successful transmission rate achieved with the proposed algorithm converges to the one obtained with the optimal centralized solution after a few iterations compared to the MAB techniques. For example, after T=30 iterations, the proposed algorithm achieves a rate of 0.99 of correctly received packets, whereas the ϵ-decreasing greedy has a rate of 0.93. It should be noted that, after a large number of iterations, the performances of the MAB algorithms in Scenario 1 converge to those in Scenario 2.

## 9. Conclusions

The autonomous power decision for NOMA schemes with a grant free access strategy has been an issue to satisfy the mMTC requirements. To the best of our knowledge, no work has been done on this problem for the MUSA scheme in order to enhance user performance with a minimal signaling overhead. In this paper, we address this issue by proposing a novel algorithm for autonomous power decision based on the proposed BEP approximation and the base station acknowledgments. Moreover, we study the efficiency of some MAB algorithms for the power allocation with two different implementation scenarios, i.e., one where the rewards of a user are only dependent on the decoding output status of its own packet and another one where they depend also on whether all users have successfully transmitted their packets or not. The proposed algorithm converges very fast to the obtained solution with a centralized resource allocation that is considered as a baseline. Moreover, the MAB algorithms have an acceptable performance, but at the cost of a larger convergence time and a higher UE complexity compared to the proposed algorithm. The latter shows the best performance with a faster convergence rate, but also with a slightly higher signaling overhead compared to the MAB algorithms, particularly for a variant propagation environment.

## Figures and Tables

**Table 1:** Quantitative comparison of the signaling overhead and the complexity at user equipment in each iteration for all algorithms. DCI, downlink control information; UCB1, upper confidence bound.

	Signaling Overhead	Complexity at UE	Power Decision
Centralized allocation	O(J·k) if k bits (depends on DCI)	O(1)	Attributed by the BS
Random selection	1 bit	O(1)	Random
Proposed algorithm	2 or 3 bits	O(1)	Iterative decision
ϵ-decreasing greedy	Scenario 1: 1 bit	O(N)	Random with ϵ probability
	Scenario 2: 2 bits		
UCB1	Scenario 1: 1 bit	O(N)	Index-based
	Scenario 2: 2 bits		
Thompson sampling	Scenario 1: 1 bit	$O\left(N\right)$	Bayesian distribution
	Scenario 2: 2 bits		

**Table 2:** Simulation settings.

Channel	AWGN with Path Losses
Users	J=12
Subcarriers	K=8
Maximum individual power	20 dBm
*N*	10 levels
Noise power	$\sigma^{2}=-14$ dBm
*T*	1000 slots
θ	0.5

## Data Availability

Not applicable.
